# Herbivorous fish (*Medialuna ancietae*) as a sustainable alternative for nutrition security in Northern Chile

**DOI:** 10.1038/s41598-021-04628-3

**Published:** 2022-01-31

**Authors:** Ivonne Lozano-Muñoz, Giorgio Castellaro, German Bueno, Jurij Wacyk

**Affiliations:** 1 Facultad de Ciencias Agronómicas, Departamento de Producción Animal, Laboratorio de Nutrición, Universidad de Chile, Santa Rosa 11315, La Pintana, CP 8820808 Santiago, Región metropolitana Chile; 2 Facultad de Recursos Naturales Renovables, Universidad Arturo Prat, Avenida Arturo Prat 2120, Iquique, Chile

**Keywords:** Nutrition, Marine biology

## Abstract

Sustainability in aquaculture is a necessity of the future, not only as the most promising means of supplying the protein that the world will require to feed its growing population but to offer needed conservation of the world’s ocean resources. The use of wild fish inputs in farm-raised fish outputs has been a primary concern of sustainability in aquaculture production. Herbivorous fish are more efficient converters of protein into fish flesh. Species of the genus *Medialuna* fish have been reported as a fast-growing, short-lived species. The native fish Acha (*Medialuna ancietae* Chirichigno 1987) in the Northern part of Chile is an over-exploited fish that has been associated with aquatic vegetation as a food source. We studied the feeding habits and nutritional composition of *M. ancietae.* For this, we developed a reference collection of marine macroalga (epidermis and nutritional composition) observed in the diet of individuals of this species for the study of digestive material. More than 90% of the components found were marine macroalgae, indicating that *M. ancietae* is an herbivorous fish. Compared to non-herbivorous fish our results showed that most of the nutrients present in the *Medialuna* diet are found at much lower levels including n-3 long-chain polyunsaturated fatty acids (49.7%) and protein (13–60%). *M. ancietae* meat provides essential components of human nutrition with a significant protein content (18.99 ± 0.26%) and 268 ± 5.9 mg/100 g of the essential n-3 long-chain polyunsaturated fatty acids. Most fed aquaculture non-herbivorous species rely on wild-captured fish for these essential nutrients, while *M. ancietae* can obtain and concentrate them from potentially cultivable macroalgae. *M. ancietae* has potential for sustainable aquaculture production as a contribution to nutrition security and the re-stocking of wild populations.

## Introduction

Aquaculture is currently the fastest growing global animal food production sector and is a key future contributor to food security; most fed aquaculture species rely on wild captured fish for essential fatty acids and micronutrients, as the fastest-growing food sector fed aquaculture demand exceeds ecological limits of these population of wild fish^1,2^. Aquaculture is the biggest global consumer of fishmeal (70%) and fish oil (73%); within the next decade, fish oil production is unlikely to meet the required quantities for aquaculture^3^. Reducing the dependence of aquaculture feeds on wild-caught fish is widely recognized as an important strategy for the sustainable growth of aquaculture^4,5^, however negative health effects can result from replacing fishmeal and fish oil with terrestrial feed ingredients (due to deficiency of certain essential nutrients) in feed for carnivorous species^5,6^ that can result in a need for the use of antimicrobials with adverse effects on the environment and human health^7^.

Feed conversion ratios (FCR) have on average improved for all species globally allowing carnivorous species to be more efficient than naturally herbivorous fish at converting feed into biomass, however, FCR do not consider nutrient retention an important aspect that reflects aquaculture's ability to deliver nutritional benefits to consumers^5^.

As a result, the use of other alternative inputs with nutritionally equivalent profiles, such as algae, will be necessary to stabilize access to fish long-chain omega-e fatty acids. The use of non-carnivorous aquaculture is also an alternative to this problem and a good strategy for sustainable aquaculture^4^. Herbivorous fishes allow for a more productive algal community by decreasing the self-shading of algae, as well as increasing the local nutrient input via herbivore excretions; herbivorous fishes can be abundant in temperate rocky-reef habitats^8^ and have been reported as short-lived and fast-growing species^9^. Herbivorous fishes have a specialized morphology of the digestive tract with the ability to assimilate seaweed compounds and to grow on a seaweed diet (stomach content with more than 50% plant material). Breakage of the algal cell wall in herbivorous fish has been attributed to lysis due to acidic stomach secretions, mechanical action, resulting from trituration gizzard-like, stomach and microbially produced enzymes^10^. The stomachs of several species of herbivorous fishes are muscular and function as gizzards to grind filamentous algae and diatoms into fine particles for chemical breakdown and subsequent absorption^11^. Herbivorous species are believed to depend on microbial fermentation to digest components of their diet, nutrients available through microbial fermentation in the form of short-chain fatty acids (SFCA)^12^. Species that fed on macroscopic algae have been associated with high levels of SCFA^13^.

In Chile, the cultivation of native marine fish is an incipient issue. Research into the cultivation of native marine fish began with capturing wild specimens, including *Merluccius australis* (Hutton 1872), *Cilus gilberti* (Abbott 1899), *Seriola lalandi* (Valenciennes 1833), *Paralichthys adpersus* (Steindachner 1867), and *Medialuna ancietae*^14^. *M. ancietae* (Acha fish) is a native species with potential for aquaculture and repopulation. *M. ancietae* lives mainly in the waters of the rocky coasts of Northern Chile and Peru and is found in wide and deep inlets, breaking from rocky beaches^15^. The catches of this species have decreased from 9000 tons per year in the 1980s to less than 100 tons per year by the middle of the 2000s. Currently, Acha fish is rarely found during spearfishing activities^16^.

*M. ancietae* is a rockfish with a high commercial value in the northern part of Chile, reaching 21 USD/kg^17^, the total landing of this fish comes exclusively from artisanal spearfishing, its consumption is offered mainly in restaurants and its meat is white with an exquisite flavor that is consistent and smooth, being able to be easily separated from the spines due to their large size and thickness. In addition, it has high regional culinary roots in Northern Chile and a high meat yield, due to the small size of its head^18^.

The local demand for the quality of its meat, and its decrease in catches make this species an interesting alternative to develop its aquaculture. *M. ancietae* is an important resource for artisanal and sport fishing in the north of Chile, but unfortunately, it shows signs of a decline in its abundance and the sizes of the fish caught^15^. Initiatives have been developed to cultivate it for commercial purposes and possible repopulation actions.

*M. ancietae* can be found with difficulty in some parts of northern Chile, especially on the coasts of the small town called Pisagua (19°36′S;70°12′W). It is characterized by the presence of large cliffs and mountain ranges, typical of the coastline of Tarapaca, Chile. The intertidal zone of this locality is conditioned by the existence of cliffs with a narrow *Lessonia berteroana* (Montagne 1842 formerly *Lessonia nigrescens*) belt that varies between 0.5 and 1.0 m. The intertidal belt of *L. nigrescens* contains 90% of the diversity of the coastal community in northern Chile, concentrating more than 70% of this in the fixation discs of this species, among the types of communities associated with fixation discs (inter-discs), nine taxa have been reported (two algae and seven invertebrates)^19^. The fauna associated with this environment mainly comprises *Fissurella* sp. and *Chiton* sp., followed by *Actinia* and *H. helianthus*, there is a specific dominance of *Fissurella* spp. (27%)^19^. The black snail *Tegula atra* (Lesson 1830) is a gastropod of the Trochidae family, which is distributed throughout the Southeast Pacific from Pacasmayo, Peru (7°24′S) to the Strait of Magellan, Chile (53°28′S). *T. atra* has been observed associated with different species of macroalgae of the order Laminariales. In northern Chile, *T. atra* is found in intertidal environments next to *Lessonia berteroana* and, in subtidal areas, next to *Lessonia trabeculata* (Villouta and Santaelices 1986)^20^.

For efficient culture and management of fish, knowledge of food and feeding habits is of striking importance, and this is intimately associated with the ecological niche they occupy in the natural environment^21^. Good reproductive performance for the successful production of juveniles is unpredictable and limiting. It has been shown that adequate nutrition and feeding of the breeders is necessary for a good quality of the egg and sperm and the production of seeds, gonadal development, and fecundity, which are affected by certain essential nutrients of the diet, especially n-3 highly unsaturated fatty acids (HUFAS). The lipid and fatty acid composition of the breeders’ diet have been identified as the main factor determining the successful reproduction and survival of the offspring. Some fish species will incorporate highly unsaturated fatty acids in the eggs, even during spawning; these HUFAS with 20 or more carbon atoms directly affect the maturation of fish and steroidogenesis through their metabolites^22,23^.

The knowledge of the feeding behaviors and the diet components are fundamental aspects to be considered when developing feeding plans that contain the profiles of these fatty acids necessary for the species to be tamed for commercial aquaculture and repopulation purposes. Part of these studies can be carried out by analyzing the digestive content with different microhistological techniques that constitute reliable procedures for identifying essential elements in herbivore feeding^24^.

This research aimed to study nutrient flow and retention in *Medialuna ancietae*, and is expected to provide a basis for *M. ancietae* environmentally sustainable aquaculture production as a contribution to nutrition security and re-stocking purposes in Northern Chile.

## Materials and methods

### Collection of fish

*M. ancietae* is an overexploited species. Therefore, its fishing is difficult, and catches are not always successful. Fishing trips were made during 2019. Fishes were caught by free diving using a modified harpoon off the shore of Pisagua (19°36′S;70°12′W), Chile. Morphometric measurements were obtained from each fish, and the stomach was extracted. All samples were transported to our laboratory under ice-storage conditions. All experiments were approved and performed according to guidelines provided by Comité de Bioseguridad y Biocustodia Universidad Arturo Prat, certificate UNAP/VRIIP Nº003/2018. This study is reported following with ARRIVE guidelines^25^.

### Collection of reference patterns and Stomach Content Analysis

Comparison patterns of the epidermis were obtained from the components commonly observed by divers in the *M. ancietae* feeding habits. The organisms were collected manually in the intertidal zone and diving off the coast of Pisagua (19°36′S; 70°12′W). and Iquique (20°13′S; 70°9′W). The macroalgae species collected were *Macrocystis integrifolia* (Bory 1826), *Lessonia berteroana*, *Corallina officinalis* var*. chilensis* (Kützing 1858), and *Glossophora kunthii* (Agardh 1882)*.*

The samples were dried in the field and fixed with 4% formalin in seawater^26^ after collection or diving. For the transfer to the laboratory in the city of Santiago, the different species of macroalgae belonging to the marine ecosystem of the area were identified. Subsequently, the epidermal tissue was removed from the macroalgae, following the diaphanization methodology^24^ and the sodium bicarbonate method^27^.

*The diaphanization method*: the material was boiled in 96% alcohol for 10 min at 150 °C, then boiled again in an aqueous solution (1:1) of 96% alcohol and 5% sodium hydroxide for another 10 min in a hot iron at 350 °C under the hood to avoid possible inflammations of alcohol and the inhalation of gases. Then, the treated material was deposited on a Petri dish and washed with distilled water until it was cleaned of reagents. Next, a solution of 5% sodium hypochlorite diluted with 50% distilled water was applied; they were allowed to stand long enough to become transparent (30 min), permanently monitoring this process. Once the material was rinsed, it was passed through distilled water five times (5 min each change) and kept in a 5% chloral hydrate solution to remove opacity. The material was kept in this solution for 10 min.

*The sodium bicarbonate method*: The material was deposited on a Petri dish, then a solution of 17.5% of sodium bicarbonate was applied, the macroalgae were soaked for 48 h, then cleared by soaking in a solution of 50% sodium hypochlorite for 20 min, and then washed with abundant distilled water. Subsequently, histological cuts (transversal) were made in the samples treated. The cuts were made manually with a scalpel, and the sample was deposited on a slide, taking care that the face to be observed is facing up, placing a drop of distilled water, and covering the sample with a 24 × 24 mm object cover. Epidermis preparations were observed under a microscope (LEICA, DM500 model equipped with ICC50W digital camera) connected to a computer to observe the epidermis on the screen of the same, photographs of all the patterns obtained using a magnification of 40× were taken to the visualization of its histological characteristics. Descriptions and illustrations of the records were made by consultation in bibliographic references^28–32^. The photographs were used for subsequent recognition of stomach contents preparations for botanical determination of their diet.

Stomach content samples were obtained immediately from eight individuals captured. The fish died at the time of capture, so it was not necessary to apply euthanasia. Stomach samples from the caught fish were weighed. Subsequently, the stomach contents were separated macroscopically by taxonomic group, and their weight was recorded. Each taxonomic group was preserved in 10% formalin. For the microhistological analysis, the material was washed with distilled water, and the diaphanization^24^ and the sodium bicarbonate^27^ method were used for the subsequent observation of the samples in an optical microscope (LEICA, DM500 model equipped with ICC50W digital camera). Images were captured for analysis. In the quantitative analysis of the food components, the gravimetric method (G) and frequency of occurrence (FO) were used. For FO, the number of stomachs containing one or more components of each food category was recorded; this number was then expressed as a percentage of all stomachs^33^. The total weight of each food category is expressed as a percentage of the overall weight of the stomach contents.

### Nutritional composition of collected macroalgae, stomach content, and M. ancietae meat

Macroalgae and *M. ancietae* were collected manually in the intertidal zone and by diving off the coasts of Pisagua (19°36′S; 70°12′W) and Iquique (20°13′S; 70°9′W). The macroalgae species collected were *Macrocystis integrifolia*, *Lessonia berteroana*, *Corallina officinalis* var. *chilensis*, and *Glosophora kunthii.*

Macroalgae and *M. ancietae* samples were immediately frozen in liquid nitrogen for transport to the laboratory and subsequent lyophilization. The lyophilized material was used for proximate and fatty acid composition following the recommended methods of the Association of Official Analytical Chemists (AOAC): fatty acid profile^34^, crude protein combustion analysis^35^ utilizing the calculation 6.25× nitrogen value, crude fat^36^, moisture content^37^, ash^38^, crude fat^36^, sodium and potassium^39^, and zinc and calcium^40^. Total carbohydrates were calculated ‟by difference”, 100% − %(crude protein + ash + crude fat + moisture).

The nutritional and fatty acid composition of the total lipid in the stomach contents were determined based on the three main components and the results of their nutritional composition.

### Data analysis

Because our data do not meet the requirements for a parametric test we performed the nonparametric Kruskal–Wallis test to compare the contribution of the different food components for each one of the study individuals using SPSS Statistics version 26 (IBM Corporation, Armonk, NY, USA). The difference was considered significant if *p* was < 0.05.

## Results

### Biological data of fishes

The biological data of collected fish are given in Table 1. Among the collected female fish, high gonadosomatic indices were present during January and September. The average weight of the fish caught was 8.964 ± 1.89 kg, with a length of 75.93 ± 6.29 cm.

**Table 1:** Biological data of Acha fish (*Medialuna ancietae*) collected in Pisagua, North of Chile (19°36'S; 70°12'W), during the year 2019.

Date	Sex	Fish weight (g)	Stomach weight (g)	Total length (cm)	Stomach content (g)	Liver weight (g)	Gonad weight (g)	ISG
January 9, 2019^1^	Female	10,909	164	83	80.70	130	480	4.40
April 25, 2019^2^	Female	11,275	118.9	80	58.57	139.19	162.41	1.44
June 11, 2019^2^	Female	8680	189.9	76	93.20	107.15	69.20	0.79
June 12, 2019^2^	Female	8540	194.8	78	95.65	100.3	65	0.76
September 8, 2019^2^	Female	10,300	159.4	82.5	76.19	108.9	544.55	5.28
November 12, 2019^1^	Male	5350	77.5	65	38.21	39.8	43.3	0.81
November 13, 2019^1^	Female	8400	180.2	70	89.42	75	254.6	3.03
November 25, 2019^1^	Female	8265	169.1	73	82.99	84.6	253.6	3.07

^1^Samples were collected in Iquique offshore (20°13'S; 70°9'W).

^2^Samples were collected in Pisagua offshore (19°36'S; 70°12'W).

The stomach weight of the collected fish was 156.65 ± 39.60 g, with a stomach content of 76.86 ± 19.48 g. On average, the stomach content (g) was 49% of the stomach weight (g) in these fish collected from January to September of 2019.

### Collection of reference patterns and stomach content analysis

**Figure 1:**
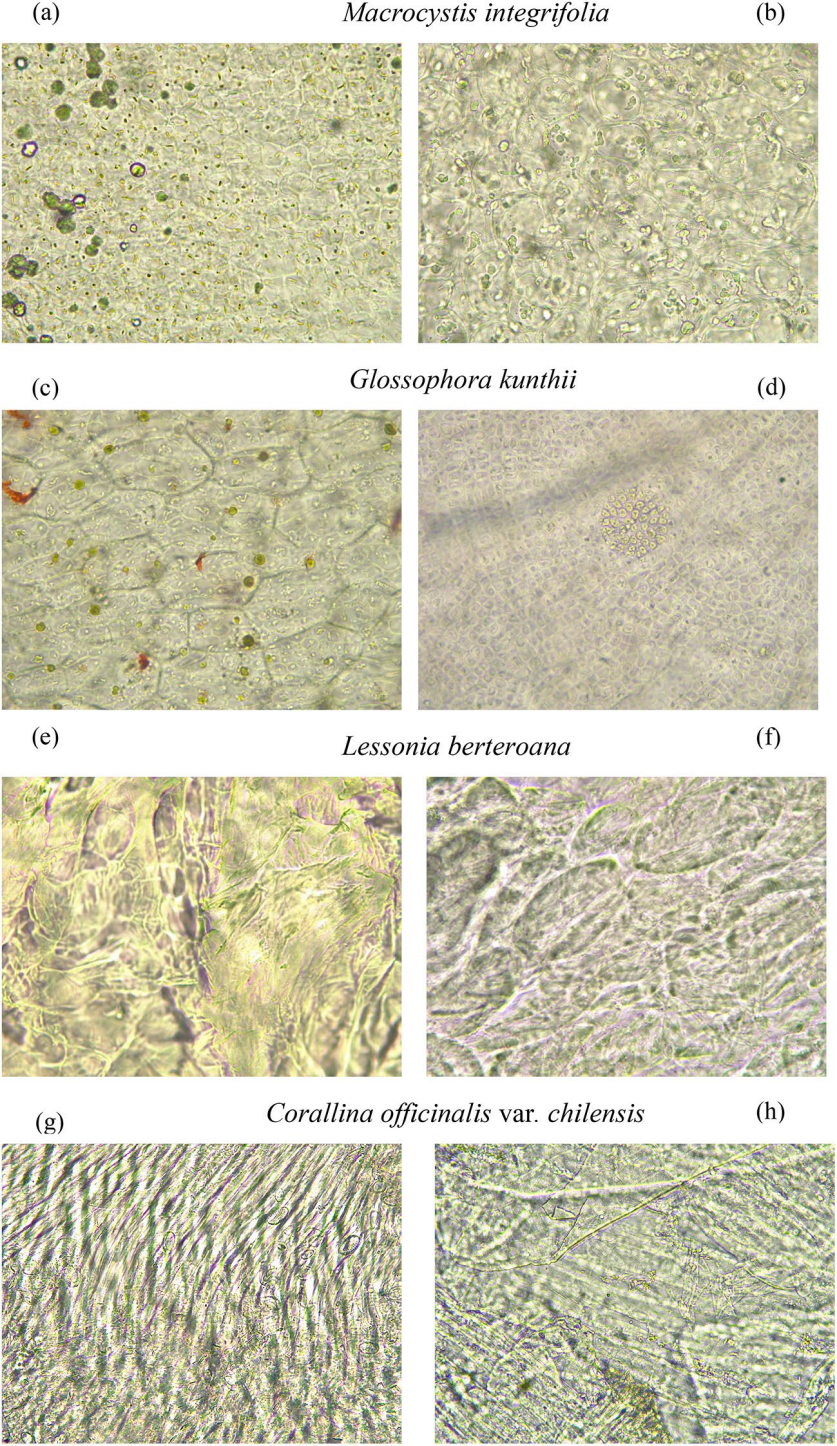
Photomicrographs (×40 magnification) of macroalgae from stomach contents of individuals of *Medialuna ancietae* prepared with sodium bicarbonate method (**a**), (**c**), (**e**), and (**g**) compared to reference slides prepared with the sodium bicarbonate method (**b**), (**d**), (**f**), and (**h**).

Remarkable differences were found when comparing reference slides prepared using the sodium bicarbonate solution method compared with the Castellaro method. The sodium bicarbonate solution maceration technique provided a much more recognizable part of the macroalgae consumed by Acha fish, including greater visibility of the different pigments present in macroalgae (Fig. 1).

**Figure 2:**
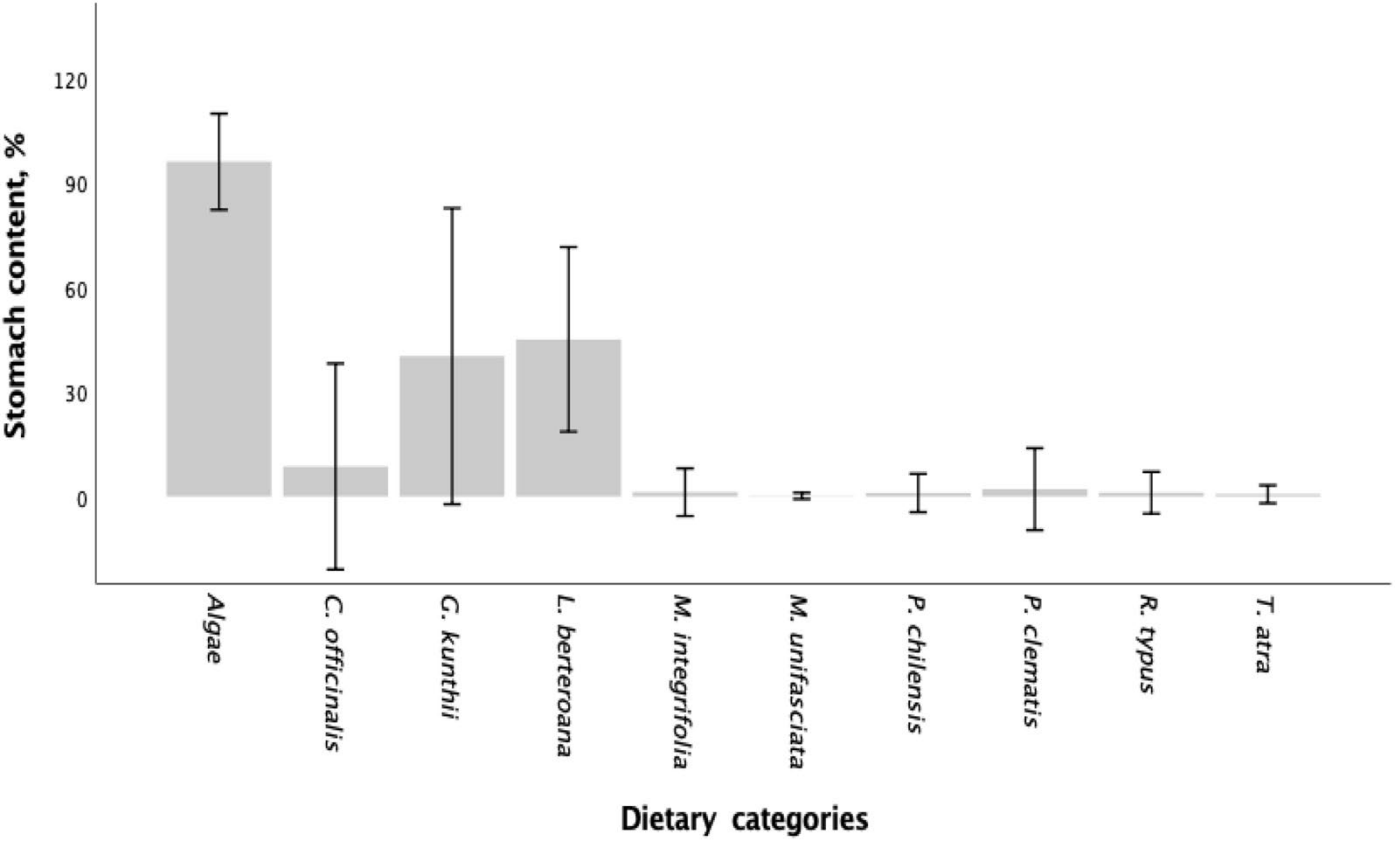
Composition of stomach content of Acha fish (*Medialuna ancietae*) collected in Pisagua, North of Chile (19°36' S; 70°12' W) during the year 2019. Broodstock diet is expressed as a gravimetric percentage (wet weight). Values are based on the mean ± SD, n = 8.

The main dietary component identified in all individuals was macroalgae, accounting for 96.08 ± 6.90%. Four different macroalgae were identified in the diet, in addition to incidental components (Fig. 2). The most abundant and consumed components by all individuals were *Lessonia berteroana* (45.08 ± 13.25 SD) followed by *Glossopohora kunthii* (40.25 ± 21.24 SD) and *Corallina officianalis* var*. chilensis* (8.57 ± 14.78 SD). *L. berteroana* and *G. kunthii* content not differed significantly (*P* < 0.05) between all individuals. *C. officianalis* was present only in the first half of the year. Only two components were present in the diet in the second half of the year: *macroalgae L. berteroana* and *G. kunthii* were identified (Table 2).

**Table 2:** Incidence of the components (wet weight, %), frequency of occurrence (FO, %) and diversity of elements per sample (DES) of the diet in the samples of stomach content of adult individuals of *Medialuna ancietae* fish collected during the year 2019 in Pisagua, North of Chile (19°36'S; 70°12'W).

Diet component	Individual 1 January 9, 2019	Individual 2 April 25, 2019	Individual 3 June 11, 2019	Individual 4 June 12, 2019	Individual 5 September 8, 2019	Individual 6 November 12, 2019	Individual 7 November 13, 2019	Individual 8 November .25, 2019	FO, %
*Corallina officinalis* var. *chilensis*^**≈**^	0	3.72	32.83	32.02	0	0	0	0	37.50
*Glossophora kunthii*	32.82	31.44	16.99	16.34	30.34	61.12	65.22	67.77	100
*Lessonia berteroana*	50.54	41.84	48.82	51.64	69.66	38.88	27.08	32.23	100
*Macrocystis integrifolia*^**≈**^	0	9.69	0	0	0	0	0	0	12.5
*% algae*	83.36	86.68	98.64	100	100	100	100	100	
*Rhynchocinetes typus*^**≈**^	0	8.49*	0	0	0	0	0	0	12.5
*Tegula atra*^**≈**^	0	3.55*	1.36*	0	0	0	0	0	25.0
*Phymactis clematis*^**≈**^	16.64	0	0	0	0	0	0	0	12.5
*Mitrela unifasciata*^**≈**^	0	1.27*	0	0	0	0	0	0	15.8
*Patria chilensis*^**≈**^	0	0	0	0	0	0	7.77*	0	12.5
DES	3	7	4	3	2	2	3	2	

*Incidental component associated with macroalga.

^**≈**^ Dietary component with significant difference between individuals. *P* < 0.05.

Among the three main components identified in the fish’s diet, high ash content was observed, with a greater presence in the red macroalgae *C. officinalis* (80.9%), the brown algae *L. berteroana* and *G.* kunthii presented a similar ash content (37% and 41%, respectively), and high carbohydrate content in both brown algae was also present (Table 3).

**Table 3:** Nutritional composition of the main categories identified in the diet of adult Acha fish (*Medialuna ancietae*). Results (except moisture) are expressed on a "dry matter" basis.

	*Lessonia berteroana*	*Glossophora kunthii*	*Corallina officinalis* var *chilensis*
Protein, %*	7.63	15.43	6.9
Moisture, %	9.06	9.1	2.7
Crude fat, %	0.53	0.48	0.2
Crude fiber	6.86	2.92	7.2
Carbohydrates, %	45.66	33.29	9.3
Ash, %	37.12	41.7	80.9
Potassium, %	12.38	7.35	0.394
Sodium, %	2.52	4.07	0.972
Zinc, %	17.4	0.124	0.108
Calcium, %	0.86	9.08	25.84

*Kjeldahl crude protein = % N × 6.25.

The brown algae showed a similar value in their fat content. The red algae *C. officinalis* showed a lower fat content (0.2%) than the brown algae. However, it showed a higher content in polyunsaturated fatty acids (51.95%) compared to the brown algae *L. berteroana* (with 32.53%) and *G. kunthii* (with 39.45%). The fatty acids with the highest presence in red algae were palmitic saturated fatty acid (16: 0) and eicosapentaenoic fatty acid (20:5n3); this red alga also showed a low content of monounsaturated fatty acids regarding the values shown for brown algae (Table 4).

**Table 4:** Fatty acid profile of the main categories identified in the diet of adult Acha fish (*Medialuna ancietae*), n-3 fatty acid values are presented in bold. Results are expressed on a "dry matter" basis.

	*Lessonia* *berteroana*	*Glossophora* *kunthii*	*Corallina* *officinalis* var*. chilensis*
Crude fat, %	0.53	0.48	0.20
Fatty acid profile (*expressed as percent of total fatty acids*)			
UKN	0.96	0.00	0.00
UKN	2.69	0.00	0.00
*Saturated fatty acids*	30.83	29.99	36.33
Lauric (12:0)	0.00	0.38	0.00
Myristic (14:0)	7.63	9.62	1.96
C15:0	0.00	0.58	0.00
Palmitic (16:0)	19.93	16.69	32.38
Margaric (17:0)	0.00	0.13	0.20
Stearic (18:0)	1.80	2.45	1.38
Arachidic (20:0)	1.07	0.14	0.04
Behenoic (22:0)	0.22	0.00	0.00
Lignoceric (24:0)	0.18	0.00	0.00
*Monounsaturated fatty acids*	23.50	30.54	11.73
Myristoleic (9c-14:1)	0.00	0.00	0.37
Palmitoleic (9c-16:1)	3.52	4.10	1.13
10c-17:1	0.69	1.08	0.25
Elaidic (9t-18:1)	0.15	0.00	0.45
Oleic (9c-18:1)	18.85	25.36	7.77
Gonodic (20:1n9)	0.29	0.00	0.81
Erucic [22:1n9]	0.00	0.00	0.77
Nervonic (24:1n9)	0.00	0.00	0.55
*Polyunsaturated fatty acids*	32.53	39.45	51.95
Linoleic (18:2n6)	4.99	15.46	3.14
**Linolenic (18:3n3)**	**2.64**	**1.45**	**0.45**
g-Linolenic [C18:3n6]	0.61	0.45	0.00
**Stearidonic (18:4n3)**	**2.95**	**0.00**	**0.00**
C20:2	0.00	1.08	0.44
**Homo-a-linolenic(20:3n3)**	**0.00**	**0.00**	**0.02**
Homo-g-linolenic [C20:3n6]	0.58	**0.00**	**0.74**
Arachidonic [20:4n6]	16.54	11.86	5.84
**EPA (20:5n3)**	**3.59**	**4.46**	**39.49**
C22:2n6	0.63	0.00	0.00
Adrenic [C22:4n6]	0.00	3.63	0.33
**DPA (22:5n3)**	**0.00**	**0.32**	**0.73**
**DHA (22:6n3)**	**0.00**	**0.74**	**0.77**

Based on the three main items identified in the diet and its nutritional composition, we found that the diet of the Acha fish is rich in carbohydrates and minerals and has a low protein content.

Among the main fatty acids present in the fish’s diet, oleic fatty acid (9c-18:1) was the most abundant. Within the polyunsaturated fatty acids, the results showed a higher presence of arachidonic fatty acid [20:4n6], followed by EPA (20:5n3) and, to a lesser extent, DHA (22:6n3). The fish meat had a low-fat content and was 18.9% protein (Table 5).

**Table 5:** Nutritional and fatty acid composition of the total lipid in meat and diet of adult specimens of Acha fish (*Medialuna ancietae*) compared with nutritional requirements for some finfish.

	Meat**	*M. ancietae* diet***	Requirement for finfish
*Protein, %	18.99 ± 0.26	10.23 ± 1.94	32–38 ^41^ 24–70 ^42^ 30-40^e^ ^43^ 40-45f.^43^
Carbohydrates, %	0	34.65 ± 3.86	12 ^41^ 20^43^
Fat, %	1.89 ± 1.68	0.45 ± 0.04	7–15 ^43^
Ash, %	1.30 ± 0.16	40.68 ± 7.40	
*Fatty acids mg/100 g*			
EPA (20:5n3)	98.05 ± 2.82	23.95 ± 9.19^≈^	200^a^; 500-750^b^ 500-2000^ g^^43^
DPA (22:5n3)	60.41 ± 2.84	0.74 ± 0.23^≈^	
DHA (22:6n3)	109.60 ± 0.28	1.5619 ± 0.61^≈^	200^a^; 500-750^b^ ^41^
EPA + DPA + DHA	268 ± 5.9	26.26 ± 8.42	
Arachidonic [20:4n6]	103.34 ± 2.34	63.39 ± 7.77	50^i^^44^
Palmitic (16:0)	457.05 ± 4.17	74.35 ± 21.98	
Oleic (9c-18:1)	512.30 ± 6.92	94.70 ± 17.72	
Linoleic (18:2n6)	21.2 ± 1.69	42.32 ± 12.75	1000^c^; 20,000^d^^41^
Linolenic (18:3n3)	11.40 ± 0.14	9.18 ± 1.22	1000^c^;800-1000^d^^41^ 500-1500^h^
Myristic (14:0)	88.01 ± 1.55	37.13 ± 6.53	

* Kjeldahl crude protein = % N × 6.25.

** Values are based on the mean ± S.D., n = 3.

*** Results are expressed on a "dry matter" basis. ^≈^ Dietary component with significant difference between individuals. *P* < 0.05. Values are based on the mean ± S.D., n = 8.

^a^Yellow tail EPA and DHA, ^b^ Channel catfish EPA and DHA, ^c^ Common carp, ^d^ Rainbow trout.

^e^Tilapia, ^f^ Trout and other marine finfish, ^g^ Marine fish, ^h^ Freshwater fish.

^i^Malabar red snapper.

## Discussion

In terms of nutritional ecology, a common problem in the study of herbivorous fishes is that their gut content is difficult to differentiate and determine its origin, hampering the classification of these species in terms of their trophic status^13^. In our study the different epidermis patterns of the macroalgae associated with the habitat of the *M. ancietae* obtained by the sodium bicarbonate technique allowed us to identify the different plant components of the *M. ancietae* diet. Within the components of the diet of the Acha fish, there is a greater presence of the algae *L. berteroana*, followed by *G. kunthii.* However, the calcareous red alga *C. officinalis* showed an important presence in the first semester of the year in females, with a low gonadosomatic index. Of the total components found in the fish’s diet, 96% correspond to macroalgae, so our results show that *M. ancietae* is an herbivorous fish^11^. Our results are consistent with studies carried out in species of the same genus, the halfmoon (*Medialuna californiensis:* Scorpididae), a herbivorous fish in the temperate waters of California^9,45^. The macroalgae identified in the diet of *M. ancietae* fish are low in fat (0.45 ± 0.04 SD), a value lower than those reported in a previous study of herbivorous fish where they obtained a high value of fat (4%) for the dietary constituents of routine consumption for mature female New Zealand butterfish *Odax pullus*^45^.

Dietary components for *M. ancientae* fat showed a higher presence of polyunsaturated fatty acids (41.31%), followed by saturated fatty acids (32.38%) with a higher presence of palmitic and myristic fatty acids and to a lesser extent, monounsaturated fatty acids (21.92%), with a higher presence of oleic fatty acid. These proportions are similar to those found for fish oil with a greater presence of polyunsaturated fatty acids followed by saturated fatty acids (with a high presence of palmitic fatty acid) and concerning the monounsaturated ones with a greater presence of oleic fatty acid^46^. The polyunsaturated fatty acid with a higher presence in the components of the diet was arachidonic, follow by eicosapentaenoic and docosahexaeoic, except *C. officinalis*, which showed a high percentage of EPA. This unexpected high EPA content may be related to the presence of diatoms observed in the *C. officinalis* samples or by the presence of marine cryptophytes^47^. Our studies showed an ARA/EPA ratio of 2.64 for the *M. ancietae* fish diet; this ratio is much higher when compared to fish oil (0.15)^48^.

Carbohydrates (34.65%) and minerals (40.68%) were the most abundant nutrients in the diet of *M. ancietae*, with important contributions of calcium from *C. officinalis* and zinc from *Lessonia berteroana.* The stomach in some herbivorous fish is a powerful muscular structure (gizzard) that allows to grind the algae fibers into fine particles that facilitates the subsequent extraction of their nutrients^11^. Several herbivorous species obtain short-chain fatty acids for energy and lipid synthesis as a result of microbial fermentation in their hindgut^12^, these strategies could allow *M. ancietae* extract energy from algae.

The *M. ancietae* diet was low in protein (10.23%). The contribution of minerals was relatively higher in the summer months due to the presence of *C. officinalis*, which presented a high ash content (80.9%). Our data shows that the diet of this herbivorous species is 13–60% lower in protein than the protein requirements reported for other species^42^. Nutritional and fatty acid composition of the total lipid in the diet of adult specimens of Acha fish differ from those of commercially produced feeds or nutritional requirements reported for other marine and freshwater non-herbivorous fish species. Most of the nutrients present in the *M. ancietae* diet were found at lower levels, including EPA (20:5n3) and DHA (22:6n3)^43^. Macronutrients (total protein, carbohydrates, and lipids) and mineral values of the stomach content obtained in our study fully agree with reported values by a previous study in adult fishes of algivore species from Great Barrier Reef, Australia^49^.

Most fed aquaculture species rely on wild-captured fish for essential fatty acids^4^, while *M. ancietae* can obtain and concentrate these essential fatty acids from potentially cultivable macroalgae^50,51^. *M. ancietae* meat can provide 268 mg/100 g of the essential n-3 long-chain polyunsaturated fatty acids (EPA + DPA + DHA).

The protein content present in the meat of *M. ancietae* (18.99 ± 0.26%) is high considering the reported protein range of 8.2–23.9 g/100 g for some freshwater and marine fish. In terms of fat (1.89 ± 1.68%), *M. ancietae* is considered a lean fish^52^. Our results showed a high protein content in *M. ancietae* meat and the presence of less protein (13–60%) in their diet when compared with the protein requirements of other non-herbivorous fish species demonstrating that *M. ancietae* is an efficient converter of feed into protein with no fish feed biomass needed.

## Conclusions

Mycohistology of the stomach contents of *M. ancietae* allowed us to identify the components of the diet; this method also allowed us to obtain a reference pattern based on the epidermis of collected aquatic plants. The results of our study show that the *M. ancietae* fish of Northern Chile is an herbivore species. Adult fish of the *M. ancietae* diet consists of more than 90% of macroalgae, mostly of *Lessonia bertorana*, *Glossosphora kuntii*, and *Corallina officinalis* var. *chilensis*. *M. ancietae* eating habits are rich in carbohydrates and low in protein content. Our study showed a high mineral content in the fish’s diet, especially in the first months of the year.

Compared to non-herbivorous fish our results showed that most of the nutrients present in the *M. ancietae* diet are found at much lower levels, including EPA and DHA (49.7%) and protein (13–60%). Most fed aquaculture non-herbivorous species rely on wild-captured fish for these essential nutrients, while *M. ancietae* can obtain and concentrate them from potentially cultivable macroalgae.

The requirements for protein, fat, and long-chain polyunsaturated fatty acids for *M. ancietae* are low and completely fish-free compared with non-herbivorous fish species that have been studied. Analyses of the feeding habits of *M. ancietae* and the nutritional quality of its meat reveal that this species has potential for use in the development of sustainable aquaculture by easing pressure on existing wild fish stocks and contribute to nutrition security in North of Chile.

## Data Availability

The datasets generated during and/or analyzed during the current study are available from the corresponding author on reasonable request.
